# Acinic cell carcinoma in pregnancy: a case report and review of the literature

**DOI:** 10.1186/1752-1947-5-91

**Published:** 2011-03-04

**Authors:** Nabil N Al-Zaher, Amani A Obeid

**Affiliations:** 1 MBC 47, Department of Otolaryngology, Head & Neck Surgery and Communication Disorders, King Faisal Specialist Hospital & Research Centre, P.O. Box 3354, Riyadh 11211, Kingdom of Saudi Arabia

## Abstract

**Introduction:**

We report an observational study on the etiology and recurrence of acinic cell carcinoma of the parotid gland that seemed to be related to pregnancy. The medical literature has never reported such an association; therefore, our case report is probably the first to mention this observation.

**Case presentation:**

This report is of a 25-year-old Arabic female patient from the United Arab Emirates, who, during her first pregnancy, developed acinic cell carcinoma of the right parotid gland that was managed with surgical excision in the form of superficial parotidectomy. During her second pregnancy, which occurred four years later, she had a recurrence of the same malignant neoplasm associated with ipsilateral malignant cervical lymphadenopathy. The patient was managed with total parotidectomy and neck dissection, as well as postoperative adjuvant radiotherapy. Our observation on this particular case of acinic cell carcinoma is that the initial onset of her neoplasm was during her first pregnancy, and the recurrence of the same malignant disease was during a subsequent pregnancy. This chronologic association raised our suspicion that there might be a possible etiologic effect of pregnancy or its associated hormonal or physiologic changes or both on the pathogenesis or etiology of acinic cell carcinoma.

**Conclusion:**

Some association might exist between pregnancy and the pathogenesis or etiology of acinic cell carcinoma.

## Introduction

Acinic cell carcinoma (ACC) is a rare malignant epithelial salivary neoplasm of a ductal cell origin. It is a low-grade malignancy that most often occurs in the parotid gland and presents at a relatively younger age than other salivary gland tumors. This malignant disease shows a female predilection, and it represents the third most common epithelial malignancy of the salivary glands in adults [1].

Possible causes of ACC include previous radiation exposure [2] and familial predisposition [3,4]. Women are more apt to have this malignant neoplasm, and endogenous hormones have been reported in normal and neoplastic salivary glands, but some of the results have been conflicting. Estrogen receptors have been reported in a minority of cases of ACC, mucoepidermoid carcinoma, and salivary duct carcinoma [1]. Progesterone receptors and androgen receptors were also seen in some cases of ACC. These findings raised suspicion that some of the salivary gland neoplasms, including ACC, might be hormonally dependent, like breast carcinoma [1,5]. The medical literature that we thoroughly reviewed had no mention of effect of pregnancy on the development or the recurrence of this malignant neoplasm we observed in this case.

## Case presentation

A 25-year-old Arabic female patient from the United Arab Emirates developed a right parotid mass lesion that was otherwise completely asymptomatic and of a stable size. She had no history of head and neck radiation treatment or a personal or family history of salivary neoplasms, but she was in the second trimester of her first pregnancy. Initially, the patient did not seek medical attention, but because of the progressive increase of the size of her parotid mass lesion, she obtained a referral to a head and neck surgeon. Her medical assessment, which included cytologic studies, indicated that the mass lesion was due to an acinic cell carcinoma.

The patient was managed surgically in the form of a right superficial parotidectomy with preservation of the ipsilateral facial nerve.

**Figure 1:**
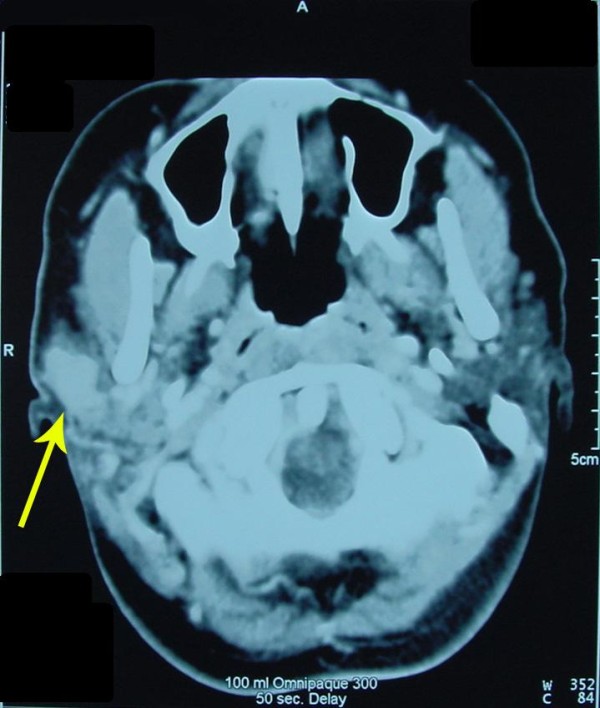
**An axial CT-scan image confirming the presence of a solid mass lesion (acinic cell carcinoma) within the right parotid gland**.

The patient remained well and had no recurrence of her malignant disease until four years later, when she became pregnant again. During the third trimester of her pregnancy, she developed a new mass lesion at the same site as her previous parotidectomy, which proved to be a recurrent acinic cell carcinoma. She also had a right submandibular malignant lymphadenopathy. The size of the recurrent acinic cell carcinoma and the ipsilateral malignant cervical lymphadenopathy progressively increased until the end of her pregnancy. The function of both facial nerves was intact. All investigations were essentially unremarkable, and no evidence of distant metastases or contraindications for surgical treatment were present. Her CT scan confirmed the presence of the recurrent mass lesion of the right parotid gland (Figure 1).

**Figure 2:**
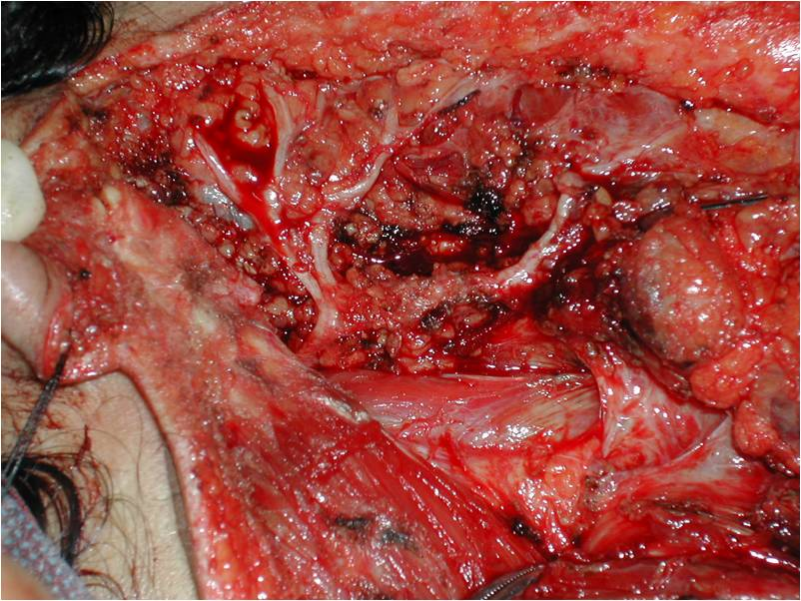
**An intraoperative view of the patient's right parotidectomy that clearly demonstrates the facial nerve that was identified and preserved intact**.

**Figure 3:**
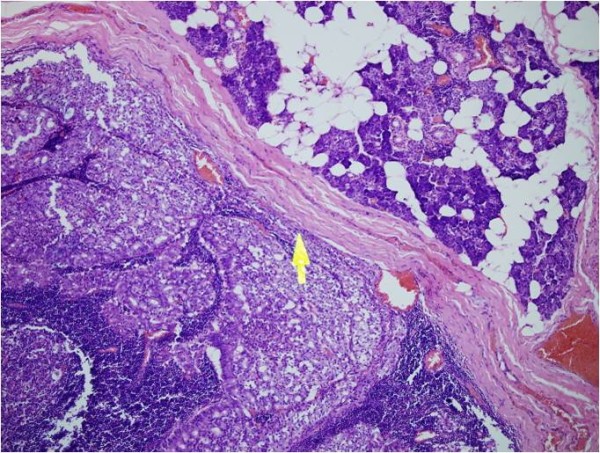
**H&E-stained pathology slide (40×)**. The neoplasm (acinic cell carcinoma) is encapsulated (arrow) and consists of clusters of malignant cells forming acini. The malignant cells themselves are pleomorphic and have atypical nuclei.

The multidisciplinary management of this patient consisted of a right total parotidectomy with preservation of the facial nerve (Figure 2) and an ipsilateral radical neck dissection followed by postoperative external-beam radiotherapy. The final histopathologic assessment confirmed the diagnosis of acinic cell carcinoma (Figure 3).

The patient recovered from her treatment and was subsequently managed with close surveillance. Her disease has been well controlled until the time of this report, and she has had no recurrence of her acinic cell carcinoma.

## Discussion

Acinic cell carcinoma (ACC) is a rare malignant epithelial salivary neoplasm of a ductal cell origin. It is a low-grade malignancy that most often occurs in the parotid gland and presents at a relatively younger age than other salivary gland tumors. This malignant disease shows a female predilection [1].

Acinic cell carcinoma constitutes approximately 17% of primary salivary gland malignancies, representing the third most common epithelial malignancy of the salivary glands in adults, and in the pediatrics age group, it is considered to be the second most common epithelial salivary malignancy after mucoepidermoid carcinoma. Women are usually more frequently diagnosed (58.8%) than men (41.2%), and according to the National Cancer Data Base Report on cancer of the head and neck, the parotid gland was the predominant site of origin (86.3%) for reported acinic cell carcinomas [5].

Possible causes of ACC include previous radiation exposure [2] and familial predisposition [3,4]. Women are more apt to have this malignant neoplasm, and endogenous hormones have been reported in normal and neoplastic salivary glands, but some of the results have been conflicting. Estrogen receptors have been reported in a minority of cases of ACC, mucoepidermoid carcinoma, and salivary duct carcinoma [1]. Progesterone receptors and androgen receptors were also seen in some cases of ACC. These findings raised suspicion that some of the salivary gland neoplasms, including ACC, might be hormonally dependent, like breast carcinoma [1,5]. The current medical literature that we thoroughly reviewed had no mention of the effect of pregnancy on the development or the recurrence of this malignant neoplasm, which we observed in this case. Interestingly, the carcinoma of this patient initially appeared during her first pregnancy, and it recurred during a subsequent pregnancy a few years later. The observed chronologic association raised our suspicion about a possible etiologic relation between pregnancy and ACC. Our observation might open doors for similar or other observations that would improve our understanding of this malignant disease and its management.

Parotid ACC typically presents with a slowly enlarging mass in the parotid region. Spiro *et al*. [6] found that 34.33% to 50.75% were palpated in the tail of the parotid gland. Pain (7.46%) and facial nerve palsy (3%) were seldom reported.

ACC has a significant tendency to recur, to produce metastases (cervical lymph nodes and lungs), and may have an aggressive evolution [7].

The genetic alterations linked to ACC of the parotid gland include alterations at chromosomes 4p, 5q, 6p, and 17p, suggesting the association of tumor-suppressor genes with the oncogenesis of these tumors [8,9].

ACC is histologically defined by serous acinar cell differentiation. However, several cell types and histomorphologic growth patterns are recognized [6,10-15].

The diagnosis of ACCs frequently presents difficulties, owing to its great radiologic [16,17] and cytologic similarity with benign tumors and with the normal acinar component of the salivary gland, respectively.

Fine-needle aspiration biopsy (FNAB) has been well established in the diagnosis of salivary gland lesions, as it provides essential information on the diagnostic and therapeutic management of these tumors; this method is highly sensitive in its diagnostic efficacy. The cytologic findings in FNABs of ACCs are usually characterized by acinar differentiated tumor cells and by certain cytoarchitectural patterns [18,19].

In addition to FNAB and other ancillary diagnostic tests, imaging studies are usually used in the pretreatment assessment and management planning of ACC, which might include ultrasonography, computed tomography, magnetic resonance imaging, and nuclear scans [17].

In general, management of ACC consists of complete surgical removal of the tumor, by total or subtotal parotidectomy, and postoperative radiotherapy may sometimes be indicated, as was the case with this patient [3,5].

The overall five-year disease-specific survival is estimated to be around 91%, and 88% at 10 years [20]. Because of the relatively high tendency of ACC to recur and to produce latent metastases, long-term follow-up is mandatory after treatment [4,5]

## Conclusion

This observational report introduces new information regarding the etiology or pathogenesis or both of acinic cell carcinoma of the salivary glands, which is expected to help in the understanding of this malignant disease and in its management, control, and prevention by surgeons and oncologists.

## Competing interests

The authors declare that they have no competing interests.

## Consent

Written informed consent was obtained from the patient for publication of this case report and the accompanying images. A copy of the written consent is available for review by the Editor-in-Chief of this journal.

## Authors' contributions

NNAZ was involved in patient evaluation and management. He is the primary author. He was the supervising surgeon of the case, and he participated in the editing of the article and in reviewing the literature. AAO was involved in data collection, literature review, and in editing the article. All authors read and approved the final manuscript.
